# Molecular brakes regulating mTORC1 activation in skeletal muscle following synergist ablation

**DOI:** 10.1152/ajpendo.00674.2013

**Published:** 2014-06-24

**Authors:** D. Lee Hamilton, Andrew Philp, Matthew G. MacKenzie, Amy Patton, Mhairi C. Towler, Iain J. Gallagher, Sue C. Bodine, Keith Baar

**Affiliations:** ^1^Health and Exercise Sciences Research Group, University of Stirling, Stirling, United Kingdom;; ^2^Division of Molecular Physiology, University of Dundee, Dundee, United Kingdom;; ^3^Neurobiology, Physiology, and Behavior, University of California Davis, Davis, California; and; ^4^Exercise and Rehabilitation Sciences, University of Birmingham, Birmingham, United Kingdom

**Keywords:** mTORC1, S6K1, AMPK, hypertrophy, skeletal muscle

## Abstract

The goal of the current work was to profile positive (mTORC1 activation, autocrine/paracrine growth factors) and negative [AMPK, unfolded protein response (UPR)] pathways that might regulate overload-induced mTORC1 (mTOR complex 1) activation with the hypothesis that a number of negative regulators of mTORC1 will be engaged during a supraphysiological model of hypertrophy. To achieve this, mTORC1-IRS-1/2 signaling, BiP/CHOP/IRE1α, and AMPK activation were determined in rat plantaris muscle following synergist ablation (SA). SA resulted in significant increases in muscle mass of ∼4% per day throughout the 21 days of the experiment. The expression of the insulin-like growth factors (IGF) were high throughout the 21st day of overload. However, IGF signaling was limited, since IRS-1 and -2 were undetectable in the overloaded muscle from *day 3* to *day 9*. The decreases in IRS-1/2 protein were paralleled by increases in GRB10 Ser^501/503^ and S6K1 Thr^389^ phosphorylation, two mTORC1 targets that can destabilize IRS proteins. PKB Ser^473^ phosphorylation was higher from 3–6 days, and this was associated with increased TSC2 Thr^939^ phosphorylation. The phosphorylation of TSC2 ^Thr1345^ (an AMPK site) was also elevated, whereas phosphorylation at the other PKB site, Thr^1462^, was unchanged at 6 days. In agreement with the phosphorylation of Thr^1345^, SA led to activation of AMPKα1 during the initial growth phase, lasting the first 9 days before returning to baseline by *day 12*. The UPR markers CHOP and BiP were elevated over the first 12 days following ablation, whereas IRE1α levels decreased. These data suggest that during supraphysiological muscle loading at least three potential molecular brakes engage to downregulate mTORC1.

skeletal muscle mass is determined by net protein balance within the muscle. During periods of growth, the rate of protein synthesis is greater than degradation (34). Load-induced increases in anabolic signals result in an increase in protein synthesis and therefore a shift toward positive protein balance. Of these anabolic signals, the activation of complex 1 of the mechanistic (mammalian) target of rapamycin (mTORC1) is a fundamental requirement (8, 12). The activity of mTORC1 is known to be regulated positively by anabolic effectors such as growth factors, amino acids, and loading and negatively by catabolic signals such as metabolic stress (14). Therefore, understanding positive and negative regulators of mTORC1 activation during a growth stimulus is essential to understanding load-induced skeletal muscle hypertrophy.

The best-studied mechanisms for activating mTORC1 and protein synthesis are via receptor tyrosine kinase activation by growth factors such as insulin-like growth factor (IGF)-I and insulin (36) and nutritional activation by branched-chain amino acids (36). Growth factors, like IGF-I, play a significant role in skeletal muscle developmental growth (9). IGF-I and MGF (mechano growth factor, the IGF-IEc splice variant) are also induced in skeletal muscle in a load-dependent manner (6, 29) and muscle-specific overexpression of IGF-I can induce significant hypertrophy (3). These data suggest that autocrine production of growth factors plays an important role in load-induced muscle growth. This hypothesis has been challenged in situ (7, 20, 21, 32), in genetic models following overload (39), following acute resistance type contractions (15, 41), and in wild-type mice following overload (30). Furthermore, recent data suggest that MGF has little role in regulating the signals that control skeletal muscle hypertrophy (10). Therefore, whether insulin or IGFs plays a significant role during in vivo load-induced hypertrophy is still equivocal.

Two other potential ways to regulate mTORC1 activity and possibly load-induced muscle hypertrophy have been described. First, the α1-catalytic subunit of AMP-activated protein kinase (AMPK) is activated by chronic overload (28). Since AMPK is known to decrease mTORC1 activity in vitro through the phosphorylation of tuberous sclerosis complex 2 (TSC2) at Thr^1345^ (24) or raptor at Ser^792^ (13), it is not surprising that knocking out AMPKα1 results in greater overload-induced hypertrophy (31). However, whether endogenous AMPK limits mTORC1 in vivo has yet to be determined. Second, endoplasmic reticulum (ER) stress through the regulation of PKB and PRAS40 could limit the activation of mTORC1 in muscle (4). However, whether ER stress is activated during overload hypertrophy and limits growth in vivo has yet to be determined.

The aim of the current study was to establish a detailed time course profile of anabolic signals (insulin/IGF-I signaling through IRS-1/2), and potential catabolic signals (ER stress and metabolic stress/AMPK activation) during overload-induced skeletal muscle hypertrophy and determine whether alterations in any of these factors are associated with altered mTORC1 activity during supraphysiological hypertrophy induced by synergist ablation. We hypothesized that a number of molecular brakes will be engaged to restrain mTORC1 activity.

## MATERIALS AND METHODS

### 

#### Ethical approval.

All procedures on Female Wistar rats (∼200 g, from Charles River Laboratories, Tranent, UK) were approved by the University of Dundee Research Ethics Committee and performed under UK Home Office project licence no. 60/3441. All surgical and collection procedures took place under inhaled anesthetic using a 2.5% concentration of isoflurane throughout. Animals were first sedated in an anesthetic induction box before placing the animal into the surgical field. Animals were allowed to recover for the appropriate time period post-stimulation and were terminated after muscle collection under anesthesia.

#### Synergist ablation.

Twenty rats were used for this study, and they were randomly assigned to five treatment groups, 3 days (*n* = 4), 6 days (*n* = 4), 9 days (*n* = 4), 12 days (*n* = 4), and 21 days (*n* = 4) of muscle hypertrophy induced by unilateral ablation of the gastrocnemius (GTN) and soleus (SOL) muscles. Animals were anesthetized, and the area above the incision was shaved and sterilized. The GTN and SOL muscles were isolated and severed at the Achilles tendon, and the distal two-thirds of the GTN and the whole SOL were removed, leaving the plantaris (PLN) intact. The overlying skin and fascia were sutured separately, and the animal was then moved to a temperature-controlled environment to recover. All animals returned to normal activity within 1 h. In the recovery period prior to collection, animals were monitored on a daily basis for signs of pain or postoperative infection. However, none of the animals showed any sign of undue discomfort or distress. On the day of collection, animals were anesthetized, and hypertrophied and contralateral control muscles were rapidly removed, snap-frozen in liquid nitrogen, and stored at −80°C until processing. Following muscle collection, animals were terminated by cervical dislocation.

#### Tissue extraction and processing.

Muscles were rapidly removed, rinsed of blood, snap-frozen in liquid nitrogen, and stored at −80°C. For processing, muscle was powdered on dry ice using a mortar and pestle and polytron homogenized in 10-fold mass excess of ice-cold sucrose lysis buffer [50 mM Tris, pH 7.5, 250 mM sucrose, 1 mM EDTA, 1 mM EGTA, 1% Triton X-100, 50 mM NaF, 1 mM NaVO_4_-Na_2_(PO_4_)_2_, and 0.1% DTT]. The homogenate was briefly vortexed and centrifuged at 4°C for 10 mins at 10,000 *g* to remove insoluble material. Protein concentrations were determined using the DC protein assay (Bio-Rad, Hercules, CA).

#### Western blotting.

Equal aliquots of protein were diluted in Laemmli sample buffer and boiled for 5 min, and then 5–10 μg of sample was subjected to SDS-PAGE on 10% acrylamide gels at a constant current equal to 20 mA per gel and transferred to Protran nitrocellulose membrane (Whatman, Dassel, Germany) using a Bio-Rad semidry transfer apparatus at 100 V for 1 h. Membranes were blocked in 5% dry milk in TBST (Tris-buffered saline + 0.1% Tween) and then incubated overnight at 4°C with appropriate primary antibody in TBST at 1:1,000. The following day, membranes were washed three times in TBST before incubation for 1 h at room temperature with peroxidase-conjugated secondary antibodies in TBST at 1:10,000 (Perbio Science, Cramlington, UK). Antibody binding was detected using an enhanced chemiluminescence HRP substrate detection kit (Millipore, Watford, UK). Imaging and band quantification were carried out using a Chemi Genius Bioimaging Gel Doc System (Syngene, Cambridge, UK). Given the rapid rate of protein accretion in the ablated muscle, appropriate loading controls proved to be an issue for the overloaded samples. Our usual loading control, eEF2 (eukaryotic elongation factor 2), changed with overload. Actin also changed substantially (data not shown). Therefore, to assess equal loading per gel, total protein content was determined via Ponceau staining or GAPDH.

#### Antibodies.

Sheep anti-IRS-2 antibody was obtained from the Division of Signal Transduction Therapy (DSTT. University of Dundee), rabbit anti-IRS-1, anti-GRB10, and anti-pGRB10 Ser^501/503^ were from Millipore (Billerica, MA), rabbit anti-S6K1 and rabbit anti-TSC2 were from Santa Cruz Biotechnology (Santa Cruz, CA), sheep anti-AMPKα1, anti-AMPKα2, and anti-phospho-(p-)TSC2 Ser^1345^ antibodies were a kind gift from Prof. Grahame Hardie (University of Dundee), rabbit-anti-p-S6K1 Thr^389^, total (t-)eEF2, p-TSC2 Thr^1642^, and p-TSC2 Thr^939^, PKB, p-PKB Thr^308^, PKB Ser^473^, p-PRAS40, t-PRAS40, and an ER stress signaling sample kit were from Cell Signaling Technologies. All other chemicals were from Sigma-Aldrich, unless stated otherwise.

#### AMPKα1 activity assay.

Approximately 50 mg of PLN powder was suspended in 100 μl of homogenization buffer [50 mM Tris·HCl, 0.25 M mannitol, 50 mM NaF, 5 mM sodium pyrophosphate, 150 mM NaCl, 5 μg/ml soybean trypsin inhibitor, 1 mM DTT, 0.1 mM PMSF, 1% (vol/vol) Triton X-100] per 20 mg of tissue and further homogenized using a hand-held polytron. The samples were left to rotate at 4°C for 30 min, and then cell debris was pelleted by centrifugation at 17,600 *g* for 5 min. The supernatants were removed and protein concentrations determined. Sheep anti-AMPKα1 antibody (5 μg) was used to immunoprecipitate AMPK from 200 μg of muscle lysate for 2 h at 4°C. The immunoprecipitates were washed in homogenization buffer and then resuspended in assay buffer [50 mM HEPES, 1 mM DTT, 0.02% (vol/vol) Brij-35]. AMP, [γ-^32^P]ATP (200 cpm/pmol), and the peptide substrate AMARAASAAALARRR were added to the immunoprecipitate at a final concentration of 200 μm. The assays were carried out for 15 min at 30°C and terminated by applying 30 μl of the reaction mixture to P81 papers (Whatman, Maidstone, UK). ATP incorporation into the peptide substrate was inhibited by placing the filter paper in 1% orthophosphoric acid. Phosphate incorporation into the peptide was measured as previously described (17).

#### Immunoprecipitations.

Rabbit TSC2 antibody (2 μg) was incubated with 1 mg of PLN lysate overnight at 4°C. The following day, TSC2 was immobilized on protein G-Sepharose for 1 h. Immunocomplexes were washed two times in sucrose lysis buffer and two times in TNE (10 mM Tris, pH 7.5, 150 mM NaCl, 1 mM EDTA, 0.1 mM Na_2_VO_4_). The immunocomplexes were resuspended in 30–50 μlof 1× Laemmli sample buffer (LSB). The samples were boiled and spun briefly to pellet the beads, and 15 μl was loaded per well for SDS-PAGE separation, and transferred as described above.

#### mRNA extraction.

Total RNA was extracted from ∼100 mg of powdered muscle using TRIzol following the manufacturer's instructions. RNA was quantified using an Ultraspec 2100 Pro spectrophotometer (Amersham Biosciences; Amersham, UK). Total RNA was then subjected to electrophoresis to check mRNA quantity and quality.

#### Reverse transcription and qPCR.

Total RNA (1 μg) was used to synthesize first-strand cDNA with AMV reverse transcriptase and oligo(DT) primers (Promega, Madison, WI) using the manufacturer's recommendations. cDNA was diluted 1:15 before use in qPCR. qPCR was performed using an Eppendorf mastercycler ep real-time thermocycler (Hamburg, Germany) along with a SYBR Green jump start *taq*-ready mix and 96-well plates (Fisher). PCR reactions were performed according to the manufacturer's instructions with the following primers: IGF-I (fwd TACTTCAACAAGCCCACAGG, rev CTCATCCACAATGCCTGTCT), IGF-II (fwd GTCGATGTTGGTGCTTCTCATC, rev GCTTGAAGGCCTGCTGAAGTAG), MGF (fwd TACTTCAACAAGCCCACAGG, rev CCTTTCCTTCTCCTTTGCAG), IRS-1 (fwd GCATCAACTTCCAGAAGCAA, rev GGAGGATTGCTGAGGTCATT), and GAPDH (fwd TGGAAAGCTGTGGCGTGAT, rev TGCTTCACCACCTTCTTGAT) used at a concentration of 10 pM. PCR conditions were as follows: initial denaturation at 95°C for 15 min followed by 40 cycles of denaturation at 94°C for 15 s, annealing at 56°C for 30 s, and extension at 72°C for 30 s. A melt curve was then performed to assess the specificity of the primers and the presence of primer dimers. PCR efficiencies were determined for each primer set. The ratio of expression of the gene of interest normalized against the standard was computed according to the equation proposed by Pfaffl et al. (33).

#### Statistics.

Data are given as means ± SE; *n* = 3 or 4 per group. For the analysis of Western blot data where different sets of animals were used across a time course but samples were paired within a time point, the data were modeled using linear mixed effects with treatment, time, or the interaction between treatment and time as a fixed effect and animal as a random effect. We used the approach of Kenward and Roger (26) to generate *P* values for these models, which is suitable for small samples. If a statistically significant main effect was found, between-level differences were assessed using Tukey contrasts. These analyses were carried out using the R statistical programming language (37) and the lme4, pbkrtest, and multcomp packages. Statistical significance was set at *P* = 0.05. The mRNA data were normalized independently for each time point. These data were analyzed using paired *t*-tests within the time point and *P* values corrected using Hochberg's step-up procedure (18).

## RESULTS

### 

#### PLN muscle growth following synergist ablation.

**Fig. 1.**
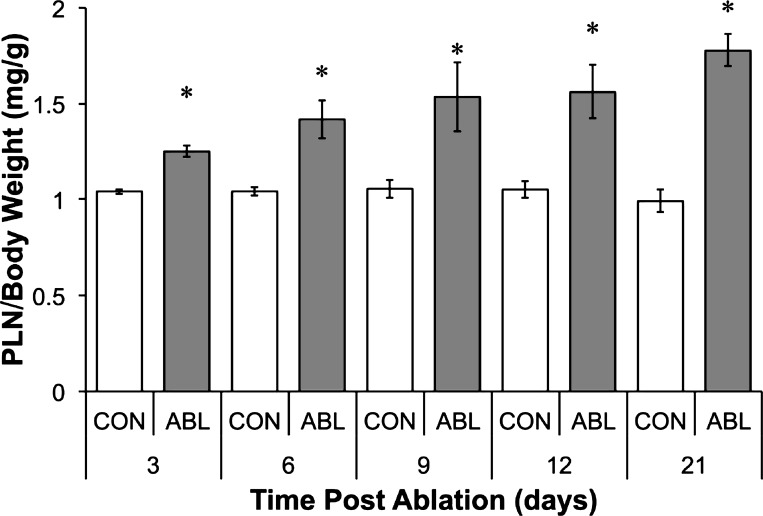
Overload-induced hypertrophy. Profile of rat plantaris (PLN) wet weight normalized to body weight following synergist ablation (ABL) of gastrocnemius and soleus muscles for 3, 6, 9, 12, or 21 days. *Significantly different from contralateral control (CON) (*P* < 0.05).

Following ablation of the SOL and GTN, the PLN muscle underwent a rapid and sustained increase in wet mass at a rate of ∼4%/day (Fig. 1). At 3 days, the wet mass of the ablated PLN was 20.3 ± 3.8% greater than the contralateral control. Steady growth was observed until the final collection point at 21 days, by which time the wet mass of the ablated PLN was 81.2 ± 12.4% greater than the contralateral control. Animal body weights were also recorded to ensure animals continued to gain weight. The animals continued to increase body weight throughout the study; therefore, the degree of muscle growth was expressed as PLN mass/body mass (Fig. 1).

#### Growth factor signaling following synergist ablation.

**Fig. 2.**
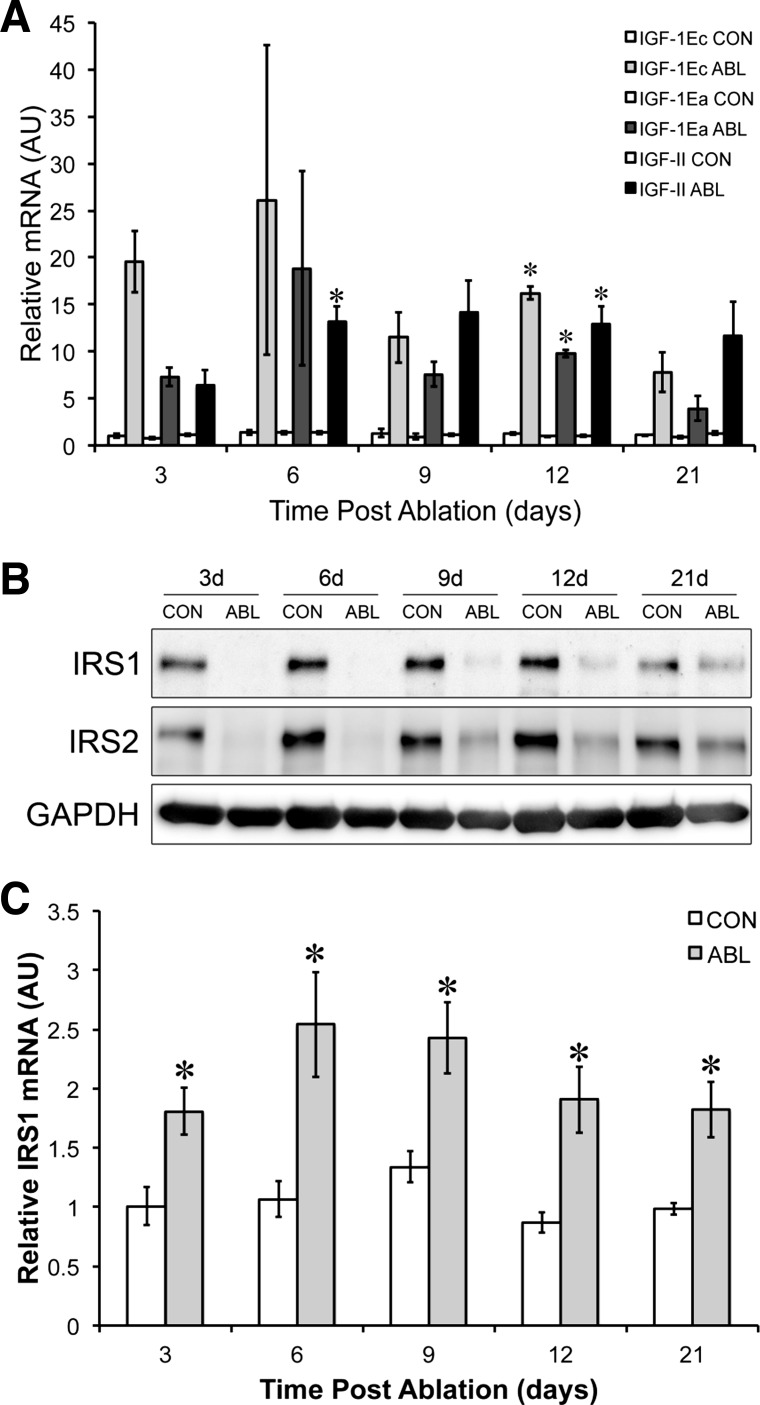
Growth factor and IRS-1/2 expression. *A*: time course of changes in IGF-1Ea/Ec and IGF-2 mRNA expression. *B*: representative blots showing IRS-1/2 protein expression with GAPDH as a loading control. *C*: time course of changes in mRNA expression of IRS-1. *Significantly different from contralateral control.

It has previously been hypothesized that overload hypertrophy is induced partly by the activation of canonical growth factor pathways (6). We therefore first measured the expression of a number of key growth factors that are known to be mechanically sensitive: IGF-I, MGF, and IGF-II. The expression of all three growth factors increased significantly at *day 12* of the overload period, while IGF-II was also significantly elevated at *day 6* (Fig. 2*A*). Next, we sought to estimate the activation of receptor tyrosine kinases by determining the association of PI3K with IRS-1/2, a crucial step in the canonical growth factor signaling cascade. At 3 days post-ablation, we could not detect PI3K association with either IRS-1 or IRS-2. We therefore instead determined the levels of IRS-1/2 following synergist ablation (Fig. 2*B*). Remarkably, both IRS-1 and -2 levels were below detection at both 3 and 6 days following synergist ablation. By *day 9*, IRS-1 and -2 protein began to return but remained well below control levels at 21 days. To better understand the control of IRS-1 expression, we assessed the impact of synergist ablation on IRS-1 mRNA levels (Fig. 2*C*). Surprisingly, IRS-1 mRNA levels were significantly elevated at all time points post-surgery (Fig. 2*C*). These data indicate either that the IRS-1 mRNA transcripts are entering a translationally inactive pool or that the degradation of IRS-1 protein far exceeds the translation of new IRS-1 from the increased transcript number.

#### S6K1 and GRB10 phosphorylation.

**Fig. 3.**
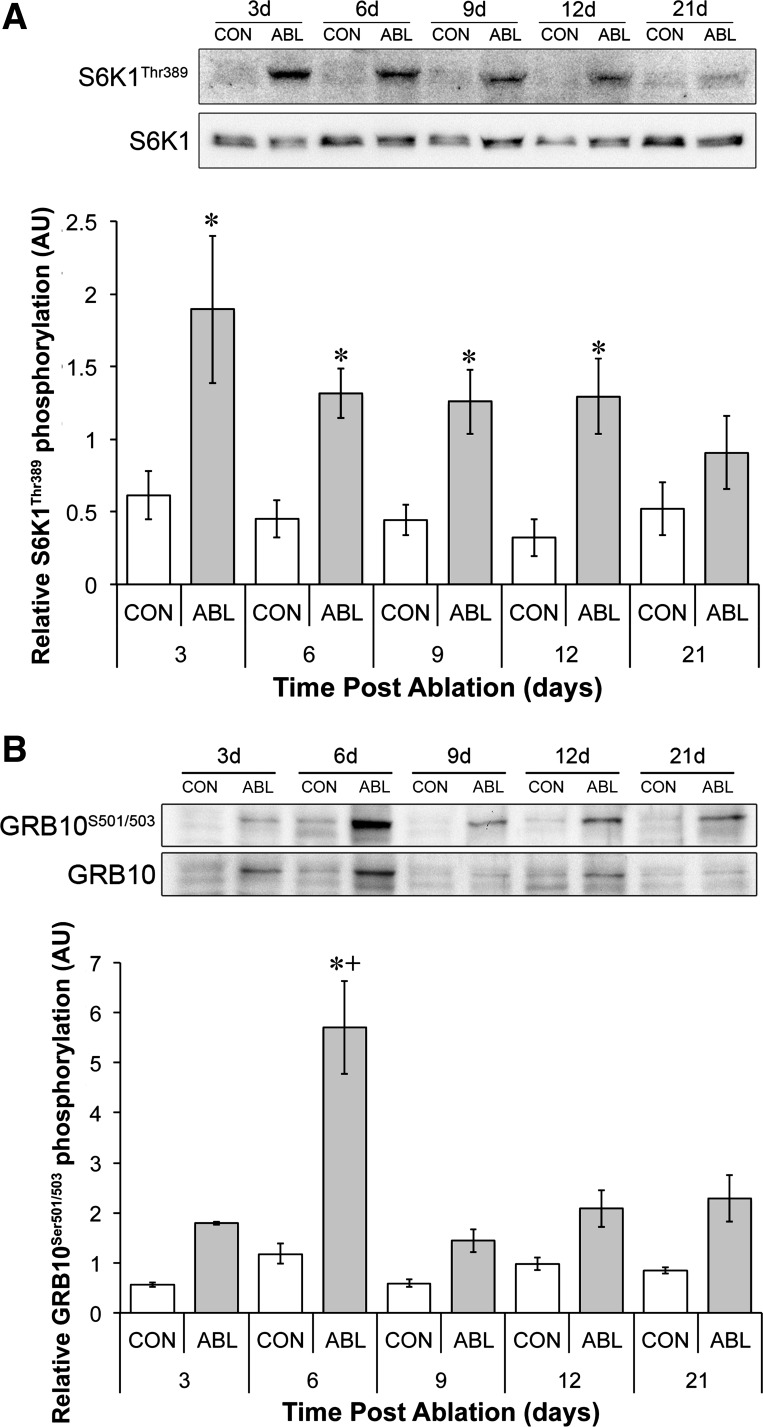
Time course in changes of S6K1 and GRB10 phosphorylation. *A*: representative S6K1 Thr^389^ and total S6K1 phosphorylation and quantification of S6K1 relative phosphorylation (ratio of phospho-S6K1 to total S6K1). *B*: representative blots showing GRB10 Ser^501/503^ and total GRB10 expression and quantification of GRB10 relative phosphorylation (ratio of phospho-GRB10 to total GRB10). *Significantly different from contralateral control (*P* < 0.05); +significantly different from all other data points (*P* < 0.05).

mTORC1 has been proposed to negatively regulate IRS-1/2 content via two mechanisms; first via S6K1 and second via GRB10. We therefore measured S6K1 Thr^389^ and GRB10 Ser^501/503^ phosphorylation over the same time period (Fig. 3*B*). S6K1 phosphorylation at Thr^389^ increased significantly up to 12 days and tended to be higher at 21 days compared with the control (3 days = 3.3 ± 0.9, 6 days = 2.3 ± 0.3, 9 days = 2.2 ± 0.4, 12 days = 2.2 ± 0.5-fold; Fig. 3*A*). This elevation in S6K1 phosphorylation occurred concomitantly with elevated GRB10 phosphorylation: GRB10 phosphorylation achieved significance at *day 6* (Fig. 3, *A* and *B*). Interestingly, the time course of peak S6K1/GRB10 phosphorylation paralleled IRS-1/2 suppression and recovery. In addition to the phosphorylation changes there was a nonsignificant trend for t-S6K1 levels to be elevated at *day 3* and a nonsignificant trend for t-GRB10 to be elevated at every time point post-ablation.

#### PKB signaling.

**Fig. 4.**
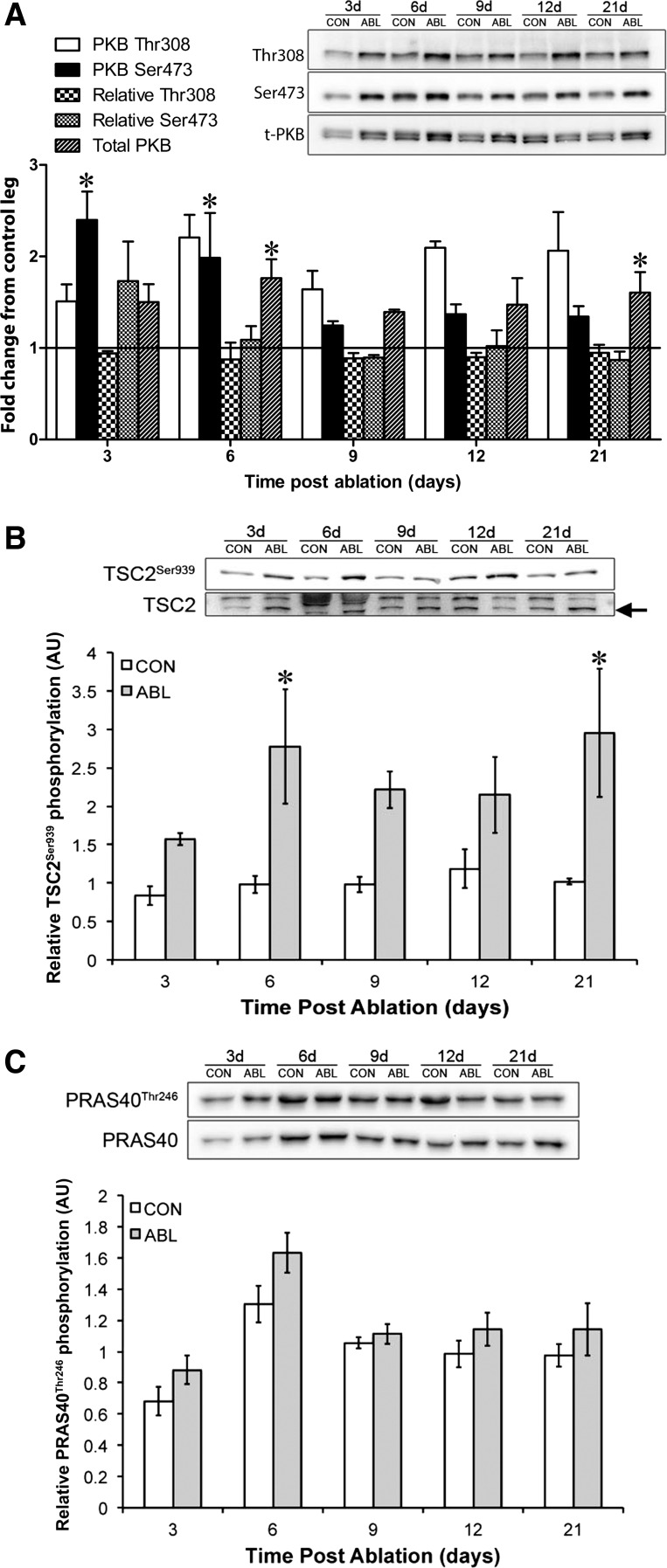
PKB signaling following synergist ablation. *A*: representative blots for PKB Thr^308^, PKB Ser^473^, and total PKB inset on a graph depicting the fold change from contralateral control for PKB Thr^308^, relative PKB Thr^308^ (ratio of phospho-PKB to total PKB), PKB Ser^473^, relative PKB Ser^473^ (ratio of phospho-PKB to total PKB), and total PKB expression throughout the time course post-ablation. *B*: representative blots for TSC2^Ser939^ and total TSC2 expression inset on a graph depicting relative phosphorylation (ratio of phospho-TSC2 to total TSC2). *C*: Representative blots for PRAS40 Ser^939^ and total PRAS40 inset on a graph depicting the relative change in PRAS40 Ser^939^ phosphorylation. *Significantly different from contralateral control (*P* < 0.05).

To estimate the time course of activation of PKB, we measured the phosphorylation of PKB at its activation sites Thr^308^ and Ser^473^ (25). In agreement with the work of Miyaziki et al. (30), we also saw significant increases in the phosphorylation of PKB 3 days following overload in rats (Fig. 4*A*). However, there was no significant change in the relative phosphorylation of PKB Thr^308^ or Ser^473^ due to significant changes in the total expression of PKB (Fig. 4*A*). We did observe increases in the phosphorylation of TSC2 at the PKB-dependent Ser^939^ phosphorylation site (23) (Fig. 4*B*), indicating that, although IRS-1/2 levels were undetectable, PKB was still capable of being activated by mechanical loading. In addition, levels of PKB protein were mechanically sensitive, increasing significantly at *days 6* and *21*. However, another target of PKB, PRAS40, did not undergo any significant changes either in phosphorylation status or in total expression.

#### AMPKα1 activity following synergist ablation.

**Fig. 5.**
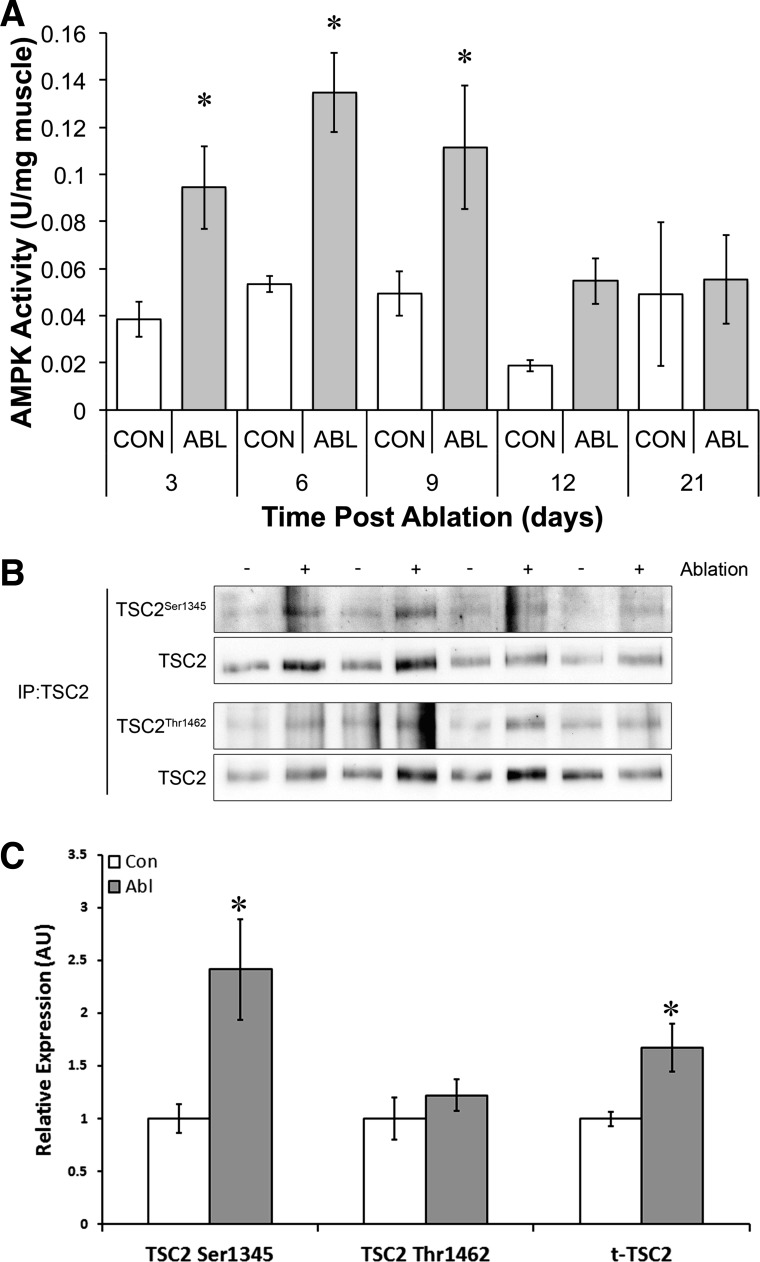
AMPK activity and signaling during overload. *A*: AMPKα1 activity following synergist ablation. *B*: representative TSC2 phospho- and total blots following immunoprecipitation (IP) after 6 days of functional overload. *C*: quantification of relative TSC2 phosphorylation (ratio phospho-TSC2 normalized to total TSC2 in immunoprecipitate) 6 days following synergist ablation. *Significantly different from contralateral control (*P* < 0.05).

To determine the time course of activation and how AMPK could decrease mTORC1 activity over a hypertrophy time course, we assayed AMPKα1 activity at 3, 6, 9, 12, and 21 days and TSC2 phosphorylation via immunoprecipitation at Ser^1345^ and Thr^1462^. AMPKα1 activity was significantly elevated at 3, 6, and 9 days (3 days = 2.26 ± 0.19-fold; 6 days = 3.22 ± 0.18-fold; and 9 days = 2.66 ± 0.28-fold) but returned to baseline by *day 12* (Fig. 5). The phosphorylation of TSC2 was determined at *day 6* when AMPKα1 activity and PKB phosphorylation peaked. The phosphorylations of Ser^1345^ and Thr^1462^ were determined since these residues are phosphorylated by AMPK and PKB, respectively (23). Consistent with previous work from our laboratory (28), the phosphorylation of TSC2 at Ser^1345^ was significantly elevated following ablation (Fig. 5*A*). However, we were unable to detect any significant change in the phosphorylation of TSC2 at Thr^1462^ (Fig. 5*B*). These data suggest that TSC1/2 may be in a more active mode following ablation and may explain the inhibitory effect of AMPKα1. Interestingly, we also saw a significant increase in the total amount of TSC2 protein (Fig. 5*C*) at this time point, indicating that increased expression of TSC2 may act as an additional brake on mTORC1 signaling in addition to the control of TSC2 phosphorylation.

#### UPR following synergist ablation.

**Fig. 6.**
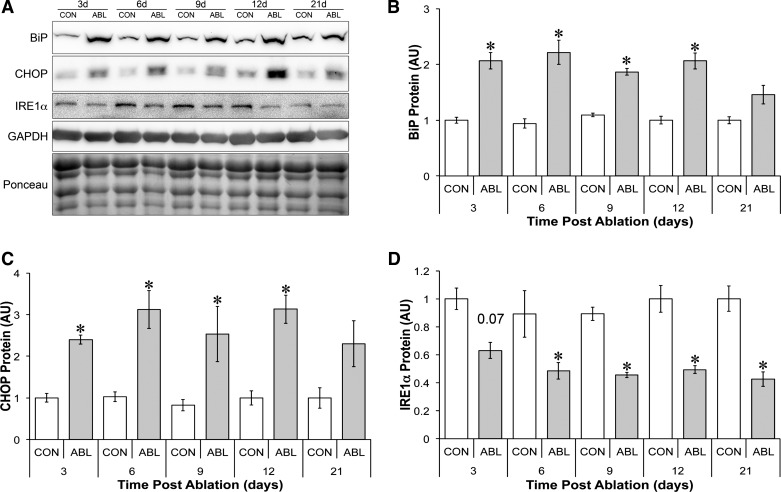
Endoplasmic reticulum (ER) stress response to synergist ablation. *A*: representative blots for ER stress markers following synergist ablation. Time course of changes in BiP (*B*), CHOP (*C*), and IRE1α protein (*D*) following synergist ablation. GAPDH expression and Ponceau staining were used to demonstrate equal loading. *Significantly different from contralateral control (*P* < 0.05).

ER stress can inhibit mTORC1 activation in muscle following high-fat feeding in mice (5), and inducers of ER stress lead to anabolic resistance in C_2_C_12_ myotubes by impairing mTORC1 activation (4). To estimate ER stress, we measured the protein chaperone BiP (B cell immunoglobulin-binding protein), the proapoptotic protein CHOP (C/EB homology protein), and the endoribonuclease inositol-requiring enzyme-1α (IRE1α). The levels of BiP, CHOP, and IRE1α were therefore measured at 3, 6, 9, 12, and 21 days post-overload. Consistent with the induction of ER stress during functional overload, the levels of BiP and CHOP were increased from 3 through 12 days of overload before returning toward control levels at 21 days (Fig. 6). IRE1α levels, however, were significantly lower, suggesting perhaps a maladaptive ER stress response (Fig. 6).

## DISCUSSION

Although it has previously been shown that IGF-I and MGF are upregulated during overload hypertrophy (6, 29) and that this correlates with an increase in signaling through mTORC1 (1), a direct relationship between these two events has not been demonstrated. In fact, Miyazaki et al. (30) have elegantly shown that PKB phosphorylation trails that of S6K1 and that the PI3K inhibitor wortmannin does not prevent the early overload-induced activation of mTORC1. Here, we add data to support that overload-induced hypertrophy in the rat PLN occurs in the absence of growth factor signaling. Despite ∼4% per day hypertrophy and increased expression of IGF-I, MGF, and IGF-II in the overloaded PLN, we saw the complete loss of IRS-1/2 protein. Such a loss of IRS-1/2 protein content would mean that signaling through the IGF-I receptor to PI3K would be significantly impaired during functional overload. The loss of IRS-1/2 protein therefore provides further evidence that mTORC1 activation during overload occurs independently of the canonical growth factor signaling pathway. These data further support the genetic data showing that a functional IGF-I receptor is not required for the activation of mTORC1 and hypertrophy following overload (39) and other work that demonstrates that PI3K is not required for loading-induced mTORC1 activation (15, 30, 41). We interpret the data to mean that during a prolonged growth stimulus IGF signaling may be decreased in a negative feedback loop to prevent excess growth. We have termed this effect a “molecular brake” on muscle hypertrophy.

The decrease in IRS-1/2 protein within the overloaded muscle could be the result of feedback inhibition downstream of mTORC1 (16). In culture, high levels of mTORC1/S6K1 activity reduce IRS-1 protein and mRNA levels, diminish its function (16), and through serine phosphorylation targets it for proteasomal degradation (43). In vivo, 18 h of extremely high S6K1 phosphorylation [up to 80-fold (35)] and activity [up to 4-fold (27)] following acute resistance type contractions do not decrease total IRS-1 or -2 levels immunoprecipated from muscle lysate (15). However, here we show that a prolonged and relatively small increase in S6K1 phosphorylation (maximum of 4-fold) is associated with complete loss of IRS-1/2. This suggests either that *1*) S6K1 activation does not drive the loss of IRS-1/2 protein; *2*) the small increase in S6K1 phosphorylation and activity in this context may have a significantly greater effect on IRS-1/2 protein, possibly due to a difference in cellular localization; or *3*) the longer duration of S6K1 activation is the key factor driving the feedback inhibition. Interestingly Harrington et al. (16) demonstrate an S6K1-dependent decrease in IRS-1 mRNA expression. Unexpectedly, however, we see significant increases in IRS-1 mRNA expression in response to SA despite the sustained increase in S6K1 phosphorylation. We suggest that either the IRS-1 mRNA transcripts enter a translationally inactive pool or that they are translationally active but IRS-1 degradation rates are so high that the total protein continues to decrease.

Concurrent with the increase in S6K1 phosphorylation, we also detected increased phosphorylation of GRB10 at the mTORC1-dependent Ser^501/503^ site (22, 42). GRB10 phosphorylation at Ser^501/503^ has recently been identified in vitro as a mechanism by which mTORC1 activation inhibits growth factor signals via destabilizing IRS function (22, 42). Ours are the first data to show that GRB10 is activated in skeletal muscle in response to a physiological signal. Furthermore, we demonstrate that the increase in GRB10 phosphorylation during SA is temporal, peaking in the first 3–6 days of growth before returning to basal levels. Despite S6K1 and GRB10 being mTORC1 substrates, the GRB10/S6K1 time courses are both very different.

Analogous to a decrease in the capacity for growth factor signaling, AMPK (24, 40) and ER stress (4) can also attenuate mTORC1 activation. Therefore, we next sought to determine the activity of these molecular brakes. As we (28) and others (31) have previously shown, the activity of AMPKα1 increases following synergist ablation. In rats, AMPKα1 activity peaks at *day 6* and returns to control levels by *day 12*. In mice, Mounier et al. (31) observed a similar peak in AMPKα1 activity at *day 7*. However, in contrast to rats, AMPKα1 activity remained high at *day 21*. This discrepancy is likely due to the different metabolic rate of the animals where the higher activity levels in the mouse results in prolonged AMPK activity. Mounier et al. also elegantly demonstrated the physiological importance of increased AMPKα1 activity: an increase in mTORC1 activity and muscle fiber hypertrophy following overload in the AMPKα1 knockout mouse. In the current study, we suggest a possible mechanism for the inhibitory effect of AMPKα1 in that the phosphorylation of TSC2 at Ser^1345^, the GTPase activating/AMPK site, was increased at *day 6*, whereas the phosphorylation of Thr^1462^, the inactivating/PKB site, was unchanged despite increased PKB phosphorylation and increased phosphorylation at the Thr^939^ site. The lack of phosphorylation of TSC2 at Thr^1462^ is in contrast to Miyazaki et al. (30), who showed that TSC2 phosphorylation at Thr^1462^ was increased at 7 days of overload. The difference between the two studies could again be a factor of rats vs. mice but is more likely due to the fact that we immunoprecipitated TSC2, whereas Miyazaki et al. used a different antibody on whole muscle lysates. We also find that the total amount of TSC2 immunoprecipitated from overloaded muscle is significantly increased, suggesting an increase in expression of TSC2 as another molecular brake on mTORC1. AMPK also feeds into mTORC1 activity by phosphorylation of Raptor at Ser^792^. We were unable to detect any alteration in Raptor phosphorylation Ser^792^ across the time course, either via standard immunoblotting or following Raptor immunoprecipitation (data not shown). This is consistent with recent work from the Hornberger laboratory demonstrating that, although phosphorylation of Raptor at alternative serine/threonine sites is an important process in mechanical activation of mTORC1, the phosphorylation status of raptor at the AMPK site Ser^792^ was detectable only when Raptor was overexpressed (11). Therefore, the question still remains as to whether AMPK regulation of mTORC1 via raptor phosphorylation is a physiologically relevant event in skeletal muscle.

Beyond the growth inhibitor AMPK and the loss of growth factor signaling through IRS-1/2, we show for the first time that overload hypertrophy activates the UPR, suggestive of ER stress. We have previously demonstrated that ER stress can block the activation of mTORC1 and limit protein synthesis (4), suggesting that ER stress is a third molecular brake that limits rapid growth in skeletal muscle following ablation. ER stress occurs when unfolded/misfolded proteins accumulate in the lumen of the ER (38). Normally, the chaperone protein BiP serves to refold damaged proteins, whereas IRE1α splices and activates XBP1 (X-box binding protein-1) and degrades mRNAs within the ER lumen (19). Both of these responses are positive adaptive changes within the cell. Spliced XBP1 serves as a transcription factor to increase the protein folding capacity of the ER (38), and degradation of ER-localized mRNA decreases the accumulation of proteins within this organelle and allows the ER to clear. Together with activating transcription factor-6 (ATF6) and protein kinase RNA (PKR)-like ER kinase (PERK), this can return ER equilibrium (38). In overloaded skeletal muscle, the levels of this integral stress response protein decreased, suggesting that the adaptive stress response in skeletal muscle is decreased by functional overload. When ER stress is prolonged and the adaptive response fails, a second, maladaptive response is initiated that can lead to apoptosis (44). Central to the maladaptive response is the transcription factor CHOP. Consistent with the growth inhibitory hypothesis, we found that CHOP levels within overloaded muscles were elevated from 3 to 21 days, suggesting that the maladaptive response was preferentially increased in response to a prolonged growth signal. However, how the maladaptive UPR could slow growth during functional overload remains to be determined. Deldicque et al. (4) do, however, shed light on a potential mechanism. They demonstrate that ER stress inducers inhibit the activation of mTORC1 by inhibiting the input of PKB on PRAS40. However, we find no significant changes in the phosphorylation of PRAS40 in response to this hypertrophy stimulus, suggesting that this negative feedback mechanism is not engaged in this scenario. Additionally, other energy stress sensors that can feed into mTORC1 such as REDD1 may also play a role in the context of overload-induced hypertrophy; however, we find no significant changes in REDD1 levels in response to overload hypertrophy (data not shown), suggesting that this is unlikely to be regulating growth in this context.

Although detailing the regulation of mTORC1 activation as reported herein is important, it is important to also consider that these data are generated in a model of supraphysiological muscle growth. We observed rates of growth of ∼4%/day. However, physiological resistance exercise paradigms tend to induce growth rates of ∼0.1%/day (2). Therefore, the next steps would be to transition into human studies to assess whether these same negative regulators of mTORC1 are engaged during a time course of hypertrophy and whether their engagement is different in muscles or individuals with different hypertrophic potential or anabolic resistance.

To conclude, overload-induced hypertrophy results in the activation of three molecular brakes that may feed back to attenuate the rate of muscle growth. First, we have demonstrated that overload-induced stress results in the loss of IRS-1/2 protein concomitant with increased S6K1 and GRB10 phosphorylation and therefore may limit the capacity for growth factors to activate mTORC1 after a meal. Second, metabolic stress results in the activation of AMPKα1 and the phosphorylation of TSC2 at Thr^1345^ in addition to an increase in total TSC2, which thereby limits the activation of mTORC1. Third, high rates of protein synthesis result in ER stress and the activation of the maladaptive unfolded protein response. The maladaptive response is epitomized by an increase in CHOP and a decrease in IRE1α, possibly decreasing protein synthesis. Together, these data suggest that the rate of muscle growth in vivo is tightly regulated even when faced with a supraphysiological stimulus such as synergist ablation. Furthermore, the loss of IRS-1/2 from overloaded muscles, likely due to GRB10 activation, supports previously described data showing that overload-induced muscle hypertrophy occurs independently of canonical growth factor signaling (30).

## GRANTS

The work was supported in part by a project grant from the Wellcome Trust (077426) to K. Baar.

## DISCLOSURES

No conflicts of interest, financial or otherwise, are declared by the author(s).

## AUTHOR CONTRIBUTIONS

Author contributions: D.L.H., A. Philp, and K.B. conception and design of research; D.L.H., A. Philp, M.G.M., A. Patton, and M.C.T. performed experiments; D.L.H., A. Philp, M.G.M., A. Patton, M.C.T., I.J.G., and K.B. analyzed data; D.L.H., A. Philp, I.J.G., S.C.B., and K.B. interpreted results of experiments; D.L.H., A. Philp, and K.B. prepared figures; D.L.H., A. Philp, and K.B. drafted manuscript; D.L.H., A. Philp, M.G.M., I.J.G., S.C.B., and K.B. edited and revised manuscript; D.L.H., A. Philp, M.G.M., A. Patton, M.C.T., I.J.G., S.C.B., and K.B. approved final version of manuscript.
